# A developmental sex difference in hippocampal neurogenesis is mediated by endogenous oestradiol

**DOI:** 10.1186/2042-6410-1-8

**Published:** 2010-11-22

**Authors:** J Michael Bowers, Jaylyn Waddell, Margaret M McCarthy

**Affiliations:** 1 Department of Physiology, University of Maryland, Baltimore School of Medicine Baltimore, MD 21201, USA; 2 Department of Psychiatry, Program in Neuroscience, University of Maryland, Baltimore School of Medicine, Baltimore, MD 21201, USA

## Abstract

**Background:**

Oestradiol is a steroid hormone that exerts extensive influence on brain development and is a powerful modulator of hippocampal structure and function. The hippocampus is a critical brain region regulating complex cognitive and emotional responses and is implicated in the aetiology of several mental health disorders, many of which exhibit some degree of sex difference. Many sex differences in the adult rat brain are determined by oestradiol action during a sensitive period of development. We had previously reported a sex difference in rates of cell genesis in the developing hippocampus of the laboratory rat. Males generate more new cells on average than females. The current study explored the effects of both exogenous and endogenous oestradiol on this sex difference.

**Methods:**

New born male and female rat pups were injected with the mitotic marker 5-bromo-2-deoxyuridine (BrdU) and oestradiol or agents that antagonize oestradiol action. The effects on cell number, proliferation, differentiation and survival were assessed at several time points. Significant differences between groups were determined by two- or thee-Way ANOVA.

**Results:**

Newborn males had higher rates of cell proliferation than females. Oestradiol treatment increased cell proliferation in neonatal females, but not males, and in the CA1 region many of these cells differentiated into neurons. The increased rate of proliferation induced by neonatal oestradiol persisted until at least 3 weeks of age, suggesting an organizational effect. Administering the aromatase inhibitor, formestane, or the oestrogen receptor antagonist, tamoxifen, significantly decreased the number of new cells in males but not females.

**Conclusion:**

Endogenous oestradiol increased the rate of cell proliferation observed in newborn males compared to females. This sex difference in neonatal neurogenesis may have implications for adult differences in learning strategy, stress responsivity or vulnerability to damage or disease.

## Introduction

Sex differences in the brain are widespread but of variable magnitude. Differences in the size of specific structures or subnuclei are well characterized, as are sex differences in the density and number of excitatory and inhibitory synapses within particular brain regions. Many sex differences in the brain are induced during a perinatal sensitive period by oestradiol following its central aromatization from testicularly derived androgen precursors (for review see [1]). The most robust neuroanatomical sex differences are found in the brain areas directly involved in reproduction, such as the preoptic area, hypothalamus and spinal cord [2-6]. The impact of steroids on these brain regions across the life-span is codified in the Organizational/Activational Hypothesis of sexual differentiation first postulated over 50 years ago [7]. Gonadal steroids, including oestradiol, also potently regulate synaptic profiles in adult brain regions not directly associated with reproduction, such as the hippocampus [8-10] and the amygdala [11].

The hippocampus subserves important behavioural and physiological functions that are influenced by sex. There are subtle, identifiable, sex differences in hippocampal volume [12] and the morphology of hippocampal cells [13-15]. There are also subtle sex differences in hippocampal associated behaviours such as spatial learning strategies, stress responsivity, and the long-term impact of negative early life events [16-20]. There are also subtle but complex sex differences in the developing hippocampus that are associated with parameters responsive to oestradiol, including calcium entry in response to depolarizing GABA [21-24] and cell genesis [25].

The hippocampus is comprised of subregions (for example, dentate gyrus (DG), CA1 and CA3) and each subregion contains distinctive cell types characterized by distinctive rates of maturation. The pyramidal cells, the principle cells of Ammon's horn, are largely formed before birth [26-29]. In contrast, granule cells, which comprise the major cell type found in the DG, are predominantly born during the first 2 weeks of postnatal life [28,30]. The development of granule cells in the molecular layer of the DG is faster in males than females [30], suggesting a sex difference in cell proliferation.

Hormonally-mediated sex differences in cell death are central to the sexual differentiation of many brain areas (for review see [31]), but less is known about sex differences in cell genesis, particularly during development. However, we recently reported that neonatal males have more new cells as indicated by 5-bromo-2-deoxyuridine (BrdU) in the DG and CA1, relative to females, with no corresponding sex difference in the number of pyknotic cells [25]. Both oestradiol and testosterone treatment increase the number of BrdU+ cells in females to the level found in males. However, whether endogenous steroids mediate the sex difference in cell proliferation is unknown. It is also unknown whether developmental steroid effects on cell proliferation are transient or organized and, as a result, endure across the lifespan. Here we report that endogenous oestradiol can generate higher rates of cell proliferation in males but plays no role in females. Consistent with previous results, treatment of females with exogenous oestradiol increases cell proliferation both acutely and 3 weeks later, suggesting that oestradiol might have an organizational role in neurogenesis. The sex difference in neurogenesis reported here may reflect a critical time period in hippocampal development that contributes to sexual dimorphisms in the adult hippocampus.

## Methods

### Animals

Newborn male and female Sprague-Dawley rats were obtained from breeder females at the University of Maryland School of Medicine. The day of birth was defined as postnatal day 0 (PN0). The animals were housed under a 12:12 h light: dark cycle, with food and water freely available. All procedures were approved by the University of Maryland School of Medicine Institutional Animal Care and Use Committee and followed National Institutes of Health Guidelines.

### Hormonal treatment of animals

**Figure 1:**
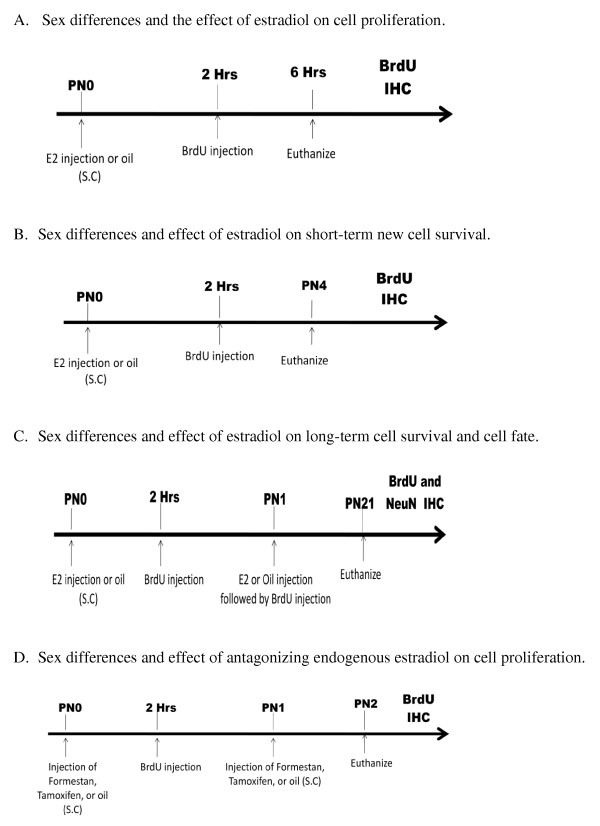
**Timeline for 5-bromo-2-deoxyuridine (BrdU) injections and hormone treatments for each experiment**. (A) Sex differences and the effects of oestradiol on cell proliferation were tested by postnatal day (PN) 0 hormone treatment with euthanasia 6 h after injection of BrdU. (B) Sex differences and the effects of oestradiol on short-term cell survival were tested by PN0 hormone treatment followed by euthanasia at PN4. (C) Sex differences and the effects of oestradiol on long-term survival and cell fate were tested by PN0 and PN1 hormone and BrdU treatments followed by euthanasia at PN21 and IHC processing for BrdU+cells. (D) Sex differences and the effects of antagonizing endogenous oestradiol were tested by treatment on PN0 and PN1 with formestane, tamoxifen or vehicle and BrdU followed by euthanasia at PN2 and IHC processing for BrdU+ cells.

On PN0, male and female rat pups were randomly distributed into different experimental groups and marked for identification by a subcutaneous (sc) ink injection in either the front or hind paws and injected sc with either oestradiol benzoate (100 μg ⁄ 0.1 mL in sesame oil), formestane (100 μg ⁄ 0.1 mL in sesame oil), tamoxifen (100 μg ⁄ 0.1 mL in sesame oil) or vehicle (sesame oil; see Figure 1 for the experimental timeline). The high dose of oestradiol is required in order to overcome the sequestering capacity of alpha-fetoprotein in the neonatal bloodstream [2] and is a dose routinely used by this laboratory to induce sexual differentiation of reproductive parameters [32]. Moreover, benzoate moiety does not alter the oestradiol's biological activity but does prolong its bioavailability. The dose of formestane has been previously used in this laboratory to decrease endogenous oestradiol in the neonatal brain to near undetectable levels [2]. The dose of tamoxifen was based on previously published literature using this drug *in vivo *[33]. Both formestane and tamoxifen readily cross the blood-brain-barrier [34,35]. Two hours after the steroid injection, all pups were injected intraperitoneally (ip) with BrdU on PN0 and PN1 (0.1 mL 0.9% sterile saline containing 100 mg ⁄ kg of BrdU), except for the experiments in which animals were injected with BrdU only on PN0. The rationale for administering BrdU 2 hours post hormone injection was based on previously published work in our laboratory in which a hormonal effect on BrdU+ cells was observed [25]. The injection sites were sealed with cyanoacrylate Vetbond Surgical Adhesive (3 M Animal Care Product, MN, USA).

### Tissue collection

Pups were deeply anaesthetized with sodium pentobarbital Fatal Plus (250 mg/kg) and transcardially perfused with 0.9% saline until there was no blood trace and then fixed with 4% paraformaldehyde (PFA). Brains were removed and fixed for 24 h in 4% PFA, followed by 48 h in 30% sucrose in PFA before being sectioned coronally on a cryostat, with each slice being 45 μm thick. Slices used for quantification were separated from each other along the rostral/caudal axis so that two contiguous sections were not analysed in the same animal. Sections were collected in series such that each animal generated 4-5 series of sections with 6-8 sections per series obtained from each brain.

### Immunohistochemistry

Free-floating tissue sections were rinsed with 0.1 M phosphate buffered saline (PBS) and then incubated with 3% hydrogen peroxide in PBS for 30 min. For BrdU immunohistochemistry, tissue sections were further incubated with 2N hydrogen chloride (HCl) for 60 min at 37°C in order to denature the DNA. After HCl incubation, the sections were rinsed with Borate buffer solution followed by PBS rinses. Finally, sections were incubated with 5% goat serum in PBS with 0.4% Triton X-100 (PBS-T) for 60 min followed by incubation in PBS-T with a monoclonal antibody against BrdU (1: 10,000, Caltag Laboratories, CA, USA) at room temperature (RT) for 60 min, then for 24 h at 4°C. Sections were rinsed in PBS and incubated with biotinylated anti-mouse secondary in PBS-T (1: 1000, Vector, Auckland, New Zealand) rinsed with PBS and incubated in Vectastain Elite ABC reagents (1: 1000, Vector). BrdU-positive cells were detected with diaminobenzidine (DAB) as chromogen, creating a dark brown colour in BrdU-positive nuclei.

For Ki-67 immunohistochemistry, tissue sections were treated as stated above with the omission of the HCl incubation step. Tissue sections were incubated with a polyclonal antibody against Ki-67 in PBS-T (1: 5000, Millipore, Darmstadt, Germany) for 60 min at RT and then 4°C for 48 h followed by biotinylated anti-rabbit secondary antibody (1: 1000). The Ki-67 positive cells were detected with DAB.

### Double-label fluorescence immunohistochemistry

Fluorescent immunohistochemistry was used to quantify the extent of colocalization of BrdU and the neuronal specific marker, NeuN. Free-floating tissue sections were rinsed with 0.1 M PBS, incubated with 3% hydrogen peroxide in PBS for 30 min, rinsed and incubated with 2 N HCl for 60 min at 37°C. After HCl incubation, sections were rinsed with Borate buffer solution followed by PBS rinses, incubated with 0.3 M glycine in 0.4% Triton X-100 (PBS-T) for 30 min, and rinsed. Sections were co-incubated with primary antibodies against anti-rat BrdU (1:500, Abcam, Cambridge, UK) and anti-mouse NeuN (1: 500, Millipore) in PBS-T, which contained 10% bovine serum albumin (BSA), for 60 min at RT and overnight at 4°C then rinsed in PBS and incubated with biotinylated anti-rat secondary (1:300, Vector) in PBS-T for 90 min followed by co-incubation with streptavidin Alexa 488 (1:1000; Invitrogen, CA, USA) and anti-mouse Alexa 568 (1:500, Invitrogen) in PBS-T for 60 min in the dark. Rinsed tissue sections were mounted onto gelatin-subbed slides and coverslipped in the dark using Vector Vectashield.

### Data analysis

**Figure 2:**
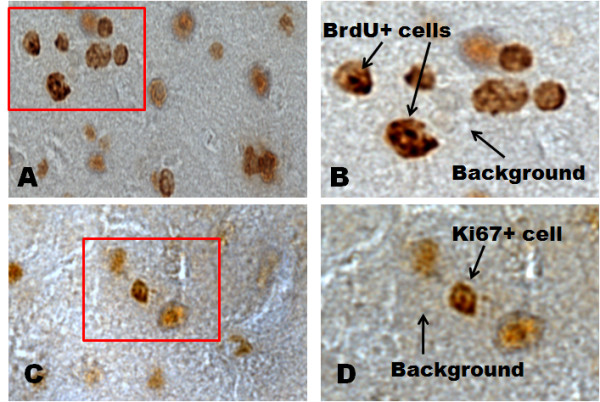
**Photomicrographs of 5-bromo-2-deoxyuridine (BrdU)+ and Ki-67+cells**. (A) An example of a BrdU+cells versus background staining at 40×. (B) 60× image of the demarked region seen in (A). (C) An example of a Ki-67+cell versus background at 40×. (D) 60× image of the demarked region seen in (C).

Each subregion of the hippocampus (CA1, CA3 and DG) was analysed using a Nikon Eclipse E600 microscope and the Neurolucida Software System (Microbrightfield, VT, USA). Cell density estimates for each subregion were determined by counting the immunopositive BrdU+, and Ki-67+, cells from the pyramidal layer of CA1 and CA3 as well as granule cell layer of the hippocampus. Cell quantification consisted of using six gridded counting frames that were within the specific subregion of the hippocampus. Each individual counting frame measured 100 × 100 μm. For all three subregions, we calculated the number of immunopositive cells that were inside the counting frames, in both the left and right hemisphere of each section. This procedure was done in four to five sections throughout the rostral hippocampus of each animal. The total number of immunopositive cells across all counting frames was averaged to give one mean value per animal per subregion. All quantifications were performed using a 40× objective. Furthermore, our cell quantification was performed with adherence to appropriate stereological principles, which included but was not limited to: (1) the presence of a nucleus; (2) immunopositive cells were of homogeneously shape; and (3) labelling intensities of immunopositive cells were distinguishable from background staining. Figure 2A-D is a photomicrograph that includes cells considered BrdU+ and Ki67+ by both investigators conducting the analyses. Lastly, the sex and hormonal condition of the animals was unknown to the investigators conducting the analyses.

For immunofluorescence, each subregion of the hippocampus was analysed using a Nikon Eclipse 80i grid confocal microscope equipped with an OptiGrid structured light source. Volocity Grid Confocal software (Improvision, Warwickshire, UK) was used to perform the analysis of Z-stacks measuring 10-12 μm collected in two channels at 0.5 μm intervals using a 100× oil objective. BrdU+ cells were selected in each subregion and the number of cells co-expressing the neuronal marker NeuN+ and BrdU+ were quantified. The immunopositive cells were counted bilaterally within each subregion, in four sections (45 μm thick), throughout the hippocampus from each animal using an 80 × 100 μm counting frame.

### Statistics

The detection of differences in the mean number of cells per region per animal between groups was determined using a three-factor ANOVA with sex, treatment and brain region as fixed factors followed by *post hoc *pairwise comparisons using *P *< 0.05 as the criterion for significance. All *post hoc *comparisons were performed using a Bonnferoni correction to control for familywise error.

## Results

### Neonatal oestradiol treatment increased BrdU+ cells in the newborn female but not the male hippocampus

**Figure 3:**
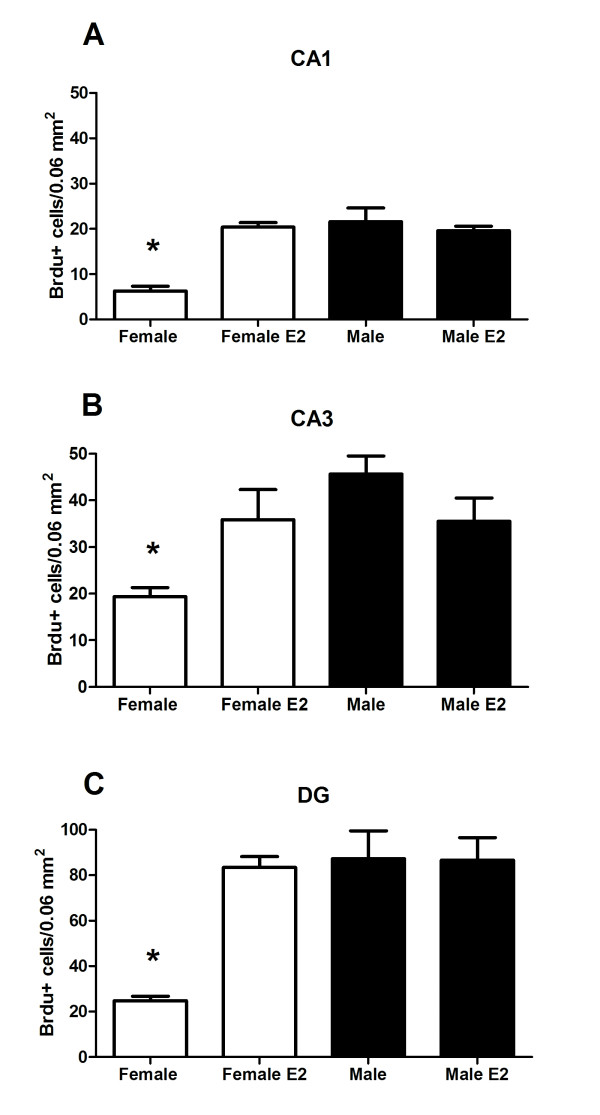
**Postnatal day (PN) 0 hormone treatment with euthanasia 6 h after injection of 5-bromo-2-deoxyuridine (BrdU)**. The mean (± standard error of mean) number of BrdU+ cells on day of birth in (A) CA1, (B) CA3 and (C) dentate gyrus. Group with the (*) symbol is significantly different from the other groups (ANOVA; *P *< 0.05).

In order to assess the effects of oestradiol on cell proliferation in developing hippocampus, pups were treated on PN0 with oestradiol followed 2 h later with a single injection of BrdU and euthanized 6 h post-BrdU injection (for procedural timeline see Figure 1-A). A 2 × 2 × 3 ANOVA indicated a significant main effect of sex [*F *(1,54) = 28.43, *P *< 0.001] confirming that males have a higher mean number of BrdU+ cells than females. There was also a main effect of treatment [*F *(1,54) = 14.68, *P *< 0.001] with oestradiol increasing the mean number of BrdU+ cells over vehicle. The three-way interaction involving sex × treatment × brain region was also significant [*F *(2,54) = 3.85, *P *< 0.03], due to the larger number of new cells being born in the DG compared to CA1 and CA3. Regardless of the total number of new cells, *post hoc *pairwise comparisons indicated vehicle treated females had significantly fewer BrdU+ cells in all three subregions of the hippocampus than either oestradiol treated females or males treated with vehicle or oestradiol (Figure 3A-C; *P *< 0.01). In females, oestradiol treatment increased the number of BrdU+ cells to the equivalent level seen in males, whereas there was no effect of oestradiol on the number of BrdU+ cells in males (*P >*0.05).

### Cells generated in the presence of oestradiol survive for both short- and long-term periods in the female hippocampus

**Figure 4:**
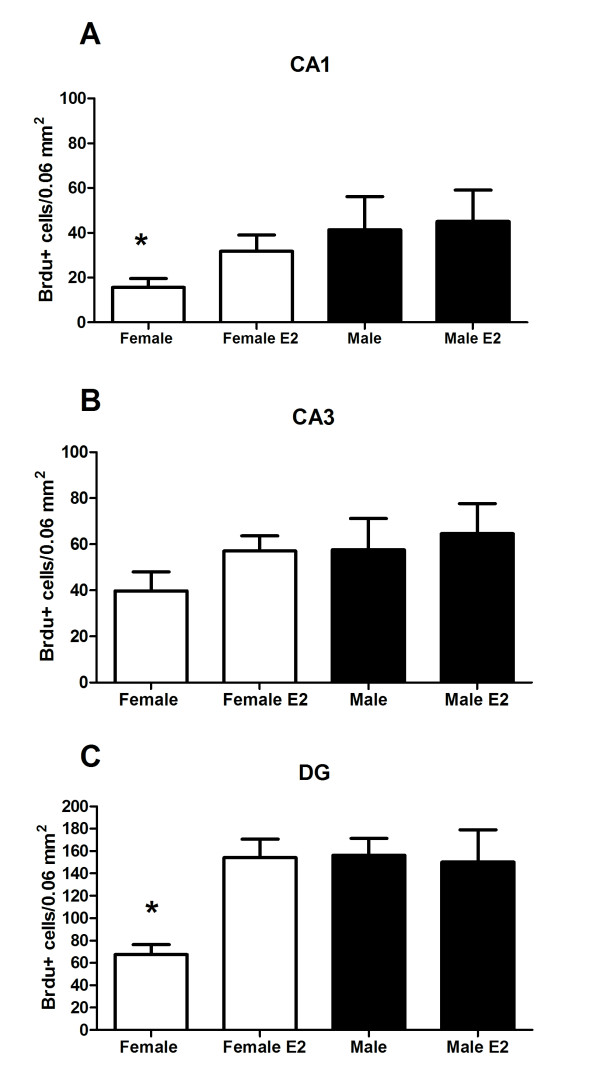
**Effects of oestradiol on short-term cell survival were tested by postnatal day (PN) 0 hormone treatment followed by euthanasia at PN4**. The mean (± standard error of mean) number of 5-bromo-2-deoxyuridine + cells on PN4 in (A) CA1, (B) CA3 and (C) dentate gyrus. Group with the (*) symbol is significantly different from the other groups (ANOVA; *P *< 0.05).

In order to assess the short- and long-term survival of newborn cells in the neonatal hippocampus, pups were treated with oestradiol followed 2 h later with a single injection of BrdU on PN0 (for procedural timeline see Figure 1-B). At PN4 (short-term period), males again had more BrdU+ cell than females [*F *(1, 57) = 7.96, *P *< 0.007] and oestradiol treatment increased the number of BrdU+ cells in DG and CA1 [*F *(1, 57) = 5.55, *P *< 0.03]. A significant three-way interaction [*F *(2,57) = 4.76, *P *< 0.03] followed by *post hoc *comparisons showed control females had fewer BrdU+ cells in both the DG and CA1 than either oestradiol treated females or males (Figure 4A-C; *Post hoc*, *P *< 0.01).

**Figure 5:**
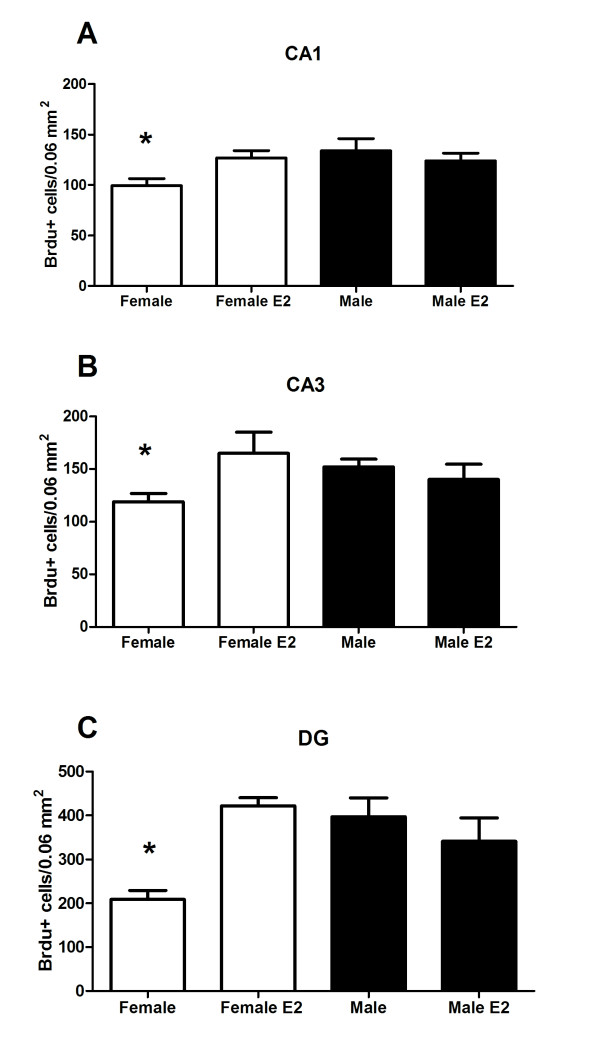
**Effects of oestradiol on long-term survival and cell fate were tested by postnatal day (PN) 0 and PN1 hormone treatments followed by euthanasia 3 weeks later**. The mean (± standard error of mean) number of 5-bromo-2-deoxyuridine + cells on PN21 in (A) CA1, (B) CA3 and (C) dentate gyrus. Group with the (*) symbol is significantly different from the other groups (ANOVA; *P *< 0.05).

In order to assess whether cells generated under the influence of oestradiol survive over a long period of time, pups were treated with oestradiol and BrdU on PN0 and PN1, then sacrificed on PN21 (for procedural timeline see Figure 1C). The same general pattern of effects observed on PN4 was again apparent on PN21, with males having more BrdU+ cells than females [*F *(1, 66) = 6.99, *P *< 0.01]; oestradiol treatment increased the number of BrdU+ cells in all three subdivisions of the hippocampus of females but not in males [*F *(2, 66) = 7.20, *P *< 0.002; Figure 5A-C, *Post hoc*, *P *< 0.01].

### The majority of cells born in the presence of oestradiol become neurons

**Figure 6:**
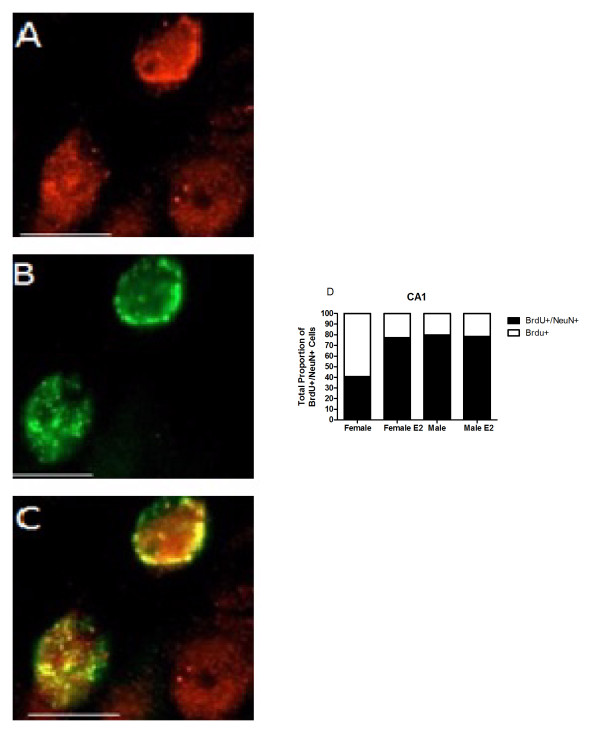
**Confocal images of neurons in CA1 pyramidal layer cell layer of the hippocampus at 100× magnification**. 5-bromo-2-deoxyuridine (BrdU) immunofluorescence was combined with immunostaining for the neuronal marker, NeuN, at 3 weeks (PN21). (A) Confocal image showing NeuN+ cells. (B) Confocal image showing BrdU+ cells. (C) Merged image of both BrdU+/NeuN+ cells. (D) Graph representing total proportion of BrdU+/NeuN+ cells in CA1. Scale bars = 10 μm.

In order to determine the fate of cells born on the first few days of postnatal life, we performed fluorescent immunohistochemistry and quantified the proportion of BrdU+ cells that are also positive for NeuN, a marker of mature neurons. The quantification of BrdU+/NeuN+ cells at PN21 indicated that both male and female groups had proportionally similar numbers of BrdU+ cells that were co-labelled with NeuN in the pyramidal cell layer of CA3 and the granular cell layer of DG (73%; ± 2% in CA3 and 81%; ± 4% in the DG). However, males had, overall, ~10% more BrdU+/NeuN+ cells than females [*F *(1, 33) = 8.73, *P *< 0.01] with the DG having the highest number of BrdU+ cells that co-labelled with NeuN in both sexes [*F *(2, 33) = 5,28, *P *< 0.05]. *Post hoc *comparison following a significant three-way interaction [*F *(2, 33) = 4.04, *P *< 0.03] revealed significantly fewer co-labelled BrdU+/NeuN+ cells in area CA1 for control females compared to either oestradiol treated females and oestradiol treated males or control males (*P *< 0.05; Figure 6). In control females, 41% of cells in CA1 born on PN0-1 were identified as neurons on PN21. In contrast, for females treated neonatally with oestradiol, 77% of the surviving cells were NeuN+, which is comparable to the 80% and 78% observed in control and oestradiol treated males, respectively.

### The sex difference in cell proliferation is still apparent at PN21 and increased in females by neonatal oestradiol treatment

**Figure 7:**
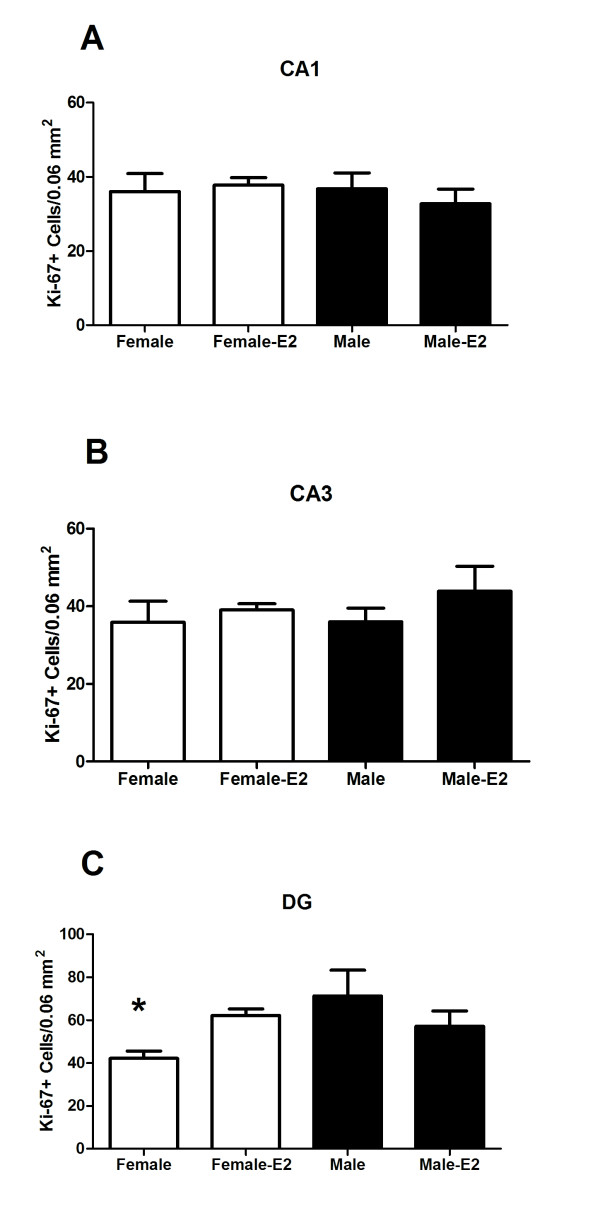
**The mean (± standard error of mean) number of Ki-67+ cells on postnatal day (PN) 21 in (A) CA1, (B) CA3 and (C) dentate gyrus of PN0 and PN1 males and females treated with oestradiol (E2) or control vehicle**. Group with the (*) symbol is significantly different from the other groups (ANOVA; *P *< 0.05).

In order to assess whether the neonatal oestradiol-induced increases in cell proliferation endure beyond the period of steroid exposure, we quantified cells expressing Ki-67, an endogenous protein that labels actively proliferating cells, on PN21. *Post hoc *comparison following a significant three-way interaction [*F *(2,36) = 4.49, *P *< 0.02] revealed that control females had significantly fewer Ki-67+ cells in the DG, as compared to control males and both neonatal oestradiol treated females and males (*P *< 0.01; Figure 7). There were no significant main effects for sex or treatment (*P's >*0.05).

### Inhibiting aromatase activity or blocking oestrogen receptor binding reduces cell proliferation in the developing male but not in the female hippocampus

**Figure 8:**
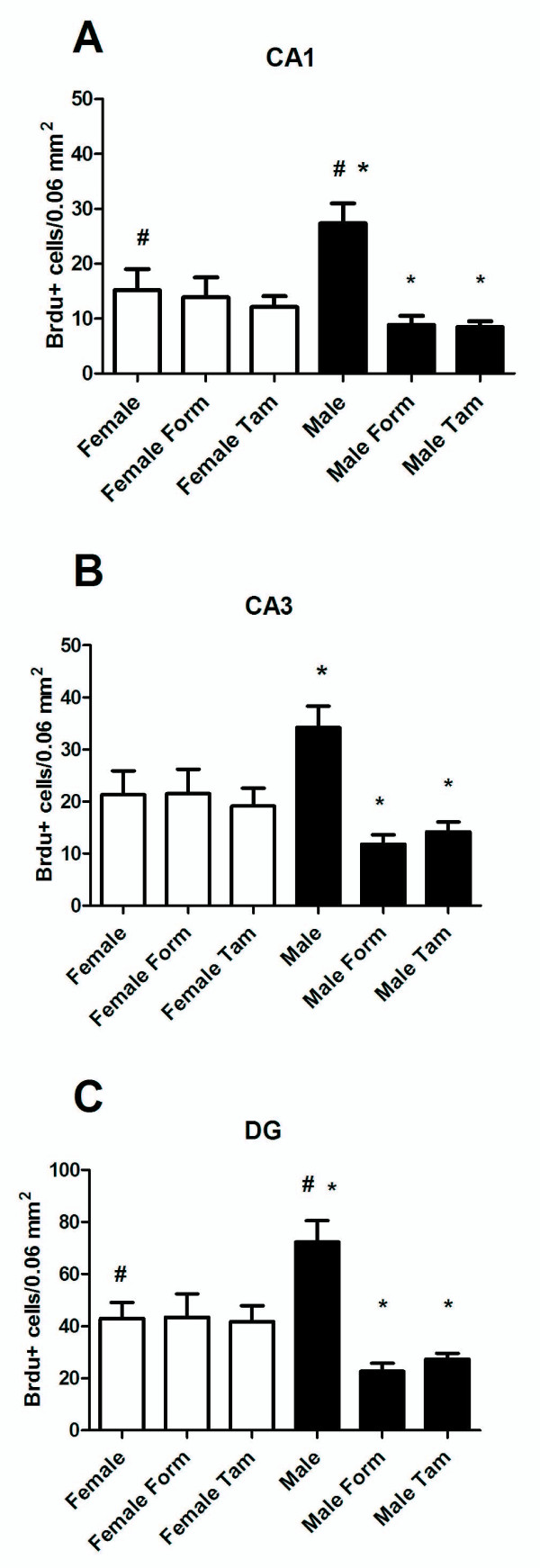
**Effects of antagonizing endogenous oestradiol were tested by treatment on postnatal day (PN) 0 and PN1 with formestane, tamoxifen or vehicle followed by euthanasia at PN2**. The mean (± standard error of mean) number of 5-bromo-2-deoxyuridine (BrdU)+ cells on PN2 in (A) CA1, (B) CA3 and (C) dentate gyrus. Females had significantly fewer BrdU+ cells compared to males (ANOVA; ^#^*P *< 0.05). Treatment with formestane and tamoxifen decreased BrdU+ cells in males, (*P < 0.01 compared with all other groups).

In order to assess the impact of endogenous oestradiol on cell proliferation, we quantified the number of BrdU+ cells in pups that were treated on PN0 and PN1 with formestane (an aromatase inhibitor), tamoxifen (an oestrogen receptor (ER) antagonist) or vehicle, followed 2 h later with an injection of BrdU (for procedural timeline see Figure 1D). Treatment with formestane or tamoxifen significantly reduced the number of BrdU+ cell compared to controls [*F*(2, 152) = 30.63, *P *< 0.001]; this effect was detected in all three subdivisions of the hippocampal complex [*F*(2, 152) = 42.54, *P *< 0.001]. *Post hoc *comparison following a significant two-way interaction of sex × treatment [*F *(2, 152) = 30.97, *P *< 0.001] indicated that the effect of formestane and tamoxifen treatment was restricted to males (*P *< 0.01; Figure 8).

## Discussion

In the present study, we found that exogenous oestradiol treatment promoted cell proliferation and survival in the neonatal female but not the male hippocampus, whereas antagonizing endogenous oestradiol synthesis or action reduced cell proliferation in the male but not in the female hippocampus. These results confirm and extend our previous report describing a sex difference and oestradiol-induced increase in the number of new cells in the neonatal hippocampus [28] by confirming a sex difference in cell genesis that is regulated by oestradiol. Our previous report included evidence of a male biased sex difference and oestradiol-induced increase in BrdU+/glial fibrillary acid (GFAP)+ expressing cells. We also quantified the number of NeuN+ cells on PN4, but did not co-label them with BrdU as there was insufficient time for neurons born on PN0 to differentiate. We found more NeuN+ cells in males, but there was no significant increase following oestradiol treatment at PN0-1. This is, again, most probably due to insufficient time for neurons born on PN0 to differentiate into neurons. Therefore our previous study did not assess neurogenesis *per se*, as opposed to the later developmental stage of PN21 used here. Moreover, many GFAP expressing cells ultimately become neurons [36] and it is possible the oestradiol-induced increase we observed in BrdU+/GFAP+ cells was a precursor to the later increase in BrdU+/NeuN+ cells seen here. The current analysis is a more accurate depiction of the fate of cells labelled at birth with BrdU under the influence of oestradiol, whereas our previous results depict the fate of new cells within a few days of birth.

There are two mechanisms by which an increase in new cell number can occur; (1) an increased rate of proliferation; and/or (2) a decreased rate of cell death. In our previous study we observed a significant increase in the number of new cells within 24 h of BrdU injection in males versus females and in females treated with oestradiol versus vehicle treated females. In the current study the same pattern was observed at the even shorter post-BrdU injection time point of 8 h. The half-life of BrdU is approximately 2 h [37] whereas the cell cycle requires approximately 24 h to complete [38]. Therefore, differences in the number of BrdU+ cells within 24 h of injection are generally interpreted as differences in the rate of cell proliferation and not cell death [37]. Our observation of more BrdU+ cells in males and oestradiol-treated females at both 8 h and 24 h post-BrdU injection is, therefore, most consistent with a hormonally mediated sex difference in cell proliferation. However, given that many new cells die shortly after being born and would not be detected here, a potential contribution of cell death to the sex differences observed cannot be entirely ruled out.

While BrdU labelling offers many advantages, such as long-term tracking of cell fate, there are also limitations associated with potential toxicity and non-specific labelling [37]. In contrast, Ki-67 is an endogenous protein that does not have any adverse effects on living cells and is expressed in all phases of the cell cycle except the resting phase and a short period at the beginning of the G1 phase [39-41]. Thus, Ki-67 is not present in quiescent and terminally differentiated cells and increased numbers of Ki-67 expressing cells is consistent with increased proliferation (but see [42]). In order to determine if sex differences or hormonal modulation of cell proliferation persisted outside of the early neonatal period we quantified the number of Ki-67 expressing cells in the hippocampus of 3-week-old animals and found males and neonatally oestradiol-treated females had more Ki-67+ cells than control females. This observation suggests a higher rate of cell proliferation was organized during the neonatal sensitive period. A precedent for the sexual differentiation of neurogenesis is evident in studies reporting differential sensitivity of adult males and females to exogenous oestradiol treatment or rates of cell birth and death in the DG. In the adult female hippocampus, oestradiol stimulates cell proliferation [43], enhances cell survival [44] and increases dendritic spine synapse density [45-48]. In contrast, the adult male hippocampus is insensitive to the spinogenesis or cell genesis inducing effects of oestradiol treatment [44,49,50]. Oestradiol effects are mediated via binding to the two oestrogen receptor isoforms, ERα and ERβ [51,52]. Both isoforms are distributed throughout the brain, including the hippocampus, and both ERs colocalize with the proliferative marker Ki-67 in the adult hippocampus [50,53,54], although, there is a region specific variation in their distribution [54-57]. Moreover, in the adult hippocampus, there are relatively low levels ERα, which is in contrast to elevated levels of ERα that occur during early postnatal development [58-60]. Nonetheless, it is unclear which receptor isoform is regulating the oestradiol's effects on cell proliferation in the developing hippocampus. Both the short-term increase in the number of BrdU cells and the higher density of Ki-67+ cells in oestradiol treated females indicate a change in proliferative rates of progenitor cells induced by oestradiol and this may be due to a change in the duration of the progenitor cell cycle. This was determined to be the case in neocortical neurogenesis [61]. Oestradiol recruits cells into the S-phase from either G1 or G0 phase, which effectively shortens the G-phase, and results in an increased rate of proliferation of dividing progenitor cells [61].

Both the amount of endogenous oestradiol and aromatase activity in the developing hippocampus are extremely low compared to the hypothalamus and do not appear to be sexually dimorphic [62], suggesting that hippocampal sensitivity to oestradiol is high and differs between the sexes, at least as indexed by hippocampal cell genesis. However, because oestradiol, the ER antagonist and the aromatase inhibitor were all administered systemically, it is possible the effects were not mediated directly at the hippocampus but, instead, were secondary to changes in other brain regions projecting to the hippocampus. Cholinergic neurons of the medial septum/diagonal band of Broca are essential for oestradiol-induced spinogenesis in adult CA1 hippocampus [63] and cholinergic input modulates maturation and integration of adult born DG granule cells [64]. Gonadally intact males release more acetylcholine into the hippocampus than females during locomotor tasks and this sex difference is organized by oestradiol during development [65,66]. The cholinergic system matures relatively early and more new septal cholinergic neurons are born in males during a brief period of gestation but the sex difference does not persist into adulthood [67]. Nonetheless, it is possible that the effects observed here are the results of oestradiol-induced acetylcholine release into the neonatal hippocampus during the early postnatal period. It is also possible that oestradiol is acting outside the central nervous system. In adults, systemic oestradiol alters the arterial cerebral blood flow in females, but not in males [68-71], via an interaction with nitric oxide [72,73]. Both ERα and ERβ, as well as the transmembrane G protein-coupled receptor, GPR30, have been identified in blood vessels [74,75] and have been implicated in the rapid vasodilator effects of oestradiol [76]. One of the most powerful stimulators of adult neurogenesis is exercise [77,78]; an effect believed to be at least partly due to enhanced blood flow and increased delivery of growth factors. Lastly, activation of the stress axis has negative effects on adult neurogenesis and the injection of BrdU and steroidal agents to neonates is undoubtedly stressful. However, given that the number of injections was carefully controlled for across groups, sex differences in stress responding can not entirely explain the current results.

Neurogenesis in the adult hippocampus is restricted to the proliferative zone of DG, with the majority of the new cells becoming granule neurons [79,80]. A notable difference in the profile of neurogenesis in the immature brain is the presence of ongoing proliferation in CA1 and CA3 of Ammon's horn. The colocalization of BrdU with NeuN in these areas indicates that the cells born early postnatally do become neurons but whether they become pyramidal neurons or interneurons is unknown. When, in development, this source of new cells is lost and neurogenesis becomes restricted to the DG is also unknown. An additional unknown is the ultimate role of these enduring neurons in the adult hippocampal function. We observed almost twice as many new cells being born in the neonatal male hippocampus compared to the female. However, when the overall size of the hippocampus is compared in males and females, either developmentally or in adulthood, the sex difference, while biased towards males, is of the order of 10%-12% [15,21]. The magnitude of the sex difference in new cells was just as strong at 3 weeks of age as when they were just a few days old. Therefore, it does not appear that these cells contribute significantly to the hippocampal volume. The early postnatal period is a time of olfactory imprinting and somatosensory stimulation from the dam. The intensity of maternal licking and grooming is greater toward male than female pups [81] and both the nature and function of olfactory learning at this time is likely to be different between the sexes. Whether the role of new neurons born during this period is related to olfactory or sensory learning remains to be determined.

## Conclusion

The hippocampus is a critical brain region involved in a variety of cognitive functions (for example, learning and memory) and both the physiologic and emotional responses to stress. Abnormalities in the hippocampus are strongly associated with affective disorders such as major depressive disorder and schizophrenia [82-85], as well as neurologic diseases such as Alzheimers. The selective vulnerability of the hippocampus to hypoxia/ischaemia following stroke, both perinatally and in adulthood [86], further emphasizes the importance of this critical brain region and the need to understand the variables that impact upon it in both males and females. Many of the sex differences observed in the adult hippocampus appear to be the result of early life events, including the impact of gonadal steroid hormones on neurogenesis during the early postnatal period.

## Abbreviations

BrdU: 5-bromo-2-deoxyuridine; DAB: diaminobenzidine; DG: dentate gyrus; ER: oestrogen receptor; GFAP: glial fibrillary acid protein; HCl: hydrogen chloride; ip: intraperitoneally; PBS: phosphate buffered saline; PFA: paraformaldehyde; PN: postnatal day; RT: room temperature; sc: subcutaneous.

## Competing interests

The authors declare that they have no competing interests.

## Authors' contributions

MB performed the immunohistochemistry, statistical analyses and drafted the manuscript. Both MB and JW treated the animals, processed the tissue samples and performed the cell counts. MM conceived the study and participated in its design and coordination. All authors read and approved the final manuscript.

## References

[B1] McCarthy MM Estradiol and the developing brain Physiol Rev 2008 88 91 124 10.1152/physrev.00010.2007 18195084 PMC2754262

[B2] Amateau SK Alt JJ Stamps CL McCarthy MM Brain estradiol content in newborn rats: sex differences, regional heterogeneity, and possible de novo synthesis by the female telencephalon Endocrinology 2004 145 2906 2917 10.1210/en.2003-1363 14988386

[B3] Mong JA McCarthy MM Steroid-induced developmental plasticity in hypothalamic astrocytes: implications for synaptic patterning J Neurobiol 1999 40 602 619 10.1002/(SICI)1097-4695(19990915)40:4<602::AID-NEU14>3.0.CO;2-O 10453059

[B4] Schwarz JM McCarthy MM Cellular mechanisms of estradiol-mediated masculinization of the brain J Steroid Biochem Mol Biol 2008 109 300 306 10.1016/j.jsbmb.2008.03.012 18430566 PMC2493288

[B5] Forger NG Rosen GJ Waters EM Jacob D Simerly RB de Vries GJ Deletion of Bax eliminates sex differences in the mouse forebrain Proc Natl Acad Sci USA 2004 101 13666 13671 10.1073/pnas.0404644101 15342910 PMC518810

[B6] Sengelaub DR Forger NG The spinal nucleus of the bulbocavernosus: firsts in androgen-dependent neural sex differences Horm Behav 2008 53 596 612 10.1016/j.yhbeh.2007.11.008 18191128 PMC2423220

[B7] Phoenix CH Goy RW Gerall AA Young WC Organizing action of prenatally administered testosterone propionate on the tissues mediating mating behavior in the female guinea pig Endocrinology 1959 65 369 382 10.1210/endo-65-3-369 14432658

[B8] Brake WG Alves SE Dunlop JC Lee SJ Bulloch K Allen PB Greengard P McEwen BS Novel target sites for estrogen action in the dorsal hippocampus: an examination of synaptic proteins Endocrinology 2001 142 1284 1289 10.1210/en.142.3.1284 11181546

[B9] Woolley CS Effects of estrogen in the CNS Curr Opin Neurobiol 1999 9 349 354 10.1016/S0959-4388(99)80051-8 10395567

[B10] Woolley CS Acute effects of estrogen on neuronal physiology Annu Rev Pharmacol Toxicol 2007 47 657 680 10.1146/annurev.pharmtox.47.120505.105219 16918306

[B11] Cooke BM Woolley CS Sexually dimorphic synaptic organization of the medial amygdala J Neurosci 2005 25 10759 10767 10.1523/JNEUROSCI.2919-05.2005 16291949 PMC6725860

[B12] Nunez JL Koss WA Juraska JM Hippocampal anatomy and water maze performance are affected by neonatal cryoanesthesia in rats of both sexes Horm Behav 2000 37 169 178 10.1006/hbeh.2000.1572 10868480

[B13] Roof RL The dentate gyrus is sexually dimorphic in prepubescent rats: testosterone plays a significant role Brain Res 1993 610 148 151 10.1016/0006-8993(93)91228-K 8518922

[B14] Roof RL Havens MD Testosterone improves maze performance and induces development of a male hippocampus in females Brain Res 1992 572 310 313 10.1016/0006-8993(92)90491-Q 1611529

[B15] Isgor C Sengelaub DR Prenatal gonadal steroids affect adult spatial behavior, CA1 and CA3 pyramidal cell morphology in rats Horm Behav 1998 34 183 198 10.1006/hbeh.1998.1477 9799628

[B16] Mirescu C Peters JD Gould E Early life experience alters response of adult neurogenesis to stress Nat Neurosci 2004 7 841 846 10.1038/nn1290 15273691

[B17] Lemaire V Koehl M Le Moal M Abrous DN Prenatal stress produces learning deficits associated with an inhibition of neurogenesis in the hippocampus Proc Natl Acad Sci USA 2000 97 11032 11037 10.1073/pnas.97.20.11032 11005874 PMC27143

[B18] Goel N Bale TL Organizational and activational effects of testosterone on masculinization of female physiological and behavioral stress responses Endocrinology 2008 149 6399 6405 10.1210/en.2008-0433 18687782 PMC2613052

[B19] Perrot-Sinal TS Kavaliers M Ossenkopp KP Spatial learning and hippocampal volume in male deer mice: relations to age, testosterone and adrenal gland weight Neuroscience 1998 86 1089 1099 10.1016/S0306-4522(98)00131-6 9697116

[B20] Bangasser DA Shors TJ The bed nucleus of the stria terminalis modulates learning after stress in masculinized but not cycling females J Neurosci 2008 28 6383 6387 10.1523/JNEUROSCI.0831-08.2008 18562608 PMC2596916

[B21] Nunez JL McCarthy MM Resting intracellular calcium concentration, depolarizing Gamma-Aminobutyric Acid and possible role of local estradiol synthesis in the developing male and female hippocampus Neuroscience 2009 158 623 634 10.1016/j.neuroscience.2008.09.061 19007865 PMC2660432

[B22] Nunez JL Bambrick LL Krueger BK McCarthy MM Prolongation and enhancement of gamma-aminobutyric acid receptor mediated excitation by chronic treatment with estradiol in developing rat hippocampal neurons Eur J Neurosci 2005 21 3251 3261 10.1111/j.1460-9568.2005.04175.x 16026463

[B23] Nunez JL McCarthy MM Androgens predispose males to GABAA-mediated excitotoxicity in the developing hippocampus Exp Neurol 2008 210 699 708 10.1016/j.expneurol.2008.01.001 18289534 PMC2430990

[B24] Nunez JL Aberdeen GW Albrecht ED McCarthy MM Impact of estradiol on gamma-aminobutyric acid- and glutamate-mediated calcium responses of fetal baboon (Papio anubis) hippocampal and cortical neurons Endocrinology 2008 149 6433 6443 10.1210/en.2007-1720 18703635 PMC2613051

[B25] Zhang JM Konkle AT Zup SL McCarthy MM Impact of sex and hormones on new cells in the developing rat hippocampus: a novel source of sex dimorphism? Eur J Neurosci 2008 27 791 800 10.1111/j.1460-9568.2008.06073.x 18333959 PMC2735768

[B26] Altman J Das GD Autoradiographic and histological evidence of postnatal hippocampal neurogenesis in rats J Comp Neurol 1965 124 319 335 10.1002/cne.901240303 5861717

[B27] Hine RJ Das GD Neuroembryogenesis in the hippocampal formation of the rat. An autoradiographic study Z Anat Entwicklungsgesch 1974 144 173 186 10.1007/BF00519773 4414797

[B28] Schlessinger AR Cowan WM Gottlieb DI An autoradiographic study of the time of origin and the pattern of granule cell migration in the dentate gyrus of the rat J Comp Neurol 1975 159 149 175 10.1002/cne.901590202 1112911

[B29] Bayer SA Development of the hippocampal region in the rat. I. Neurogenesis examined with 3H-thymidine autoradiography J Comp Neurol 1980 190 87 114 10.1002/cne.901900107 7381056

[B30] Muramatsu R Ikegaya Y Matsuki N Koyama R Neonatally born granule cells numerically dominate adult mice dentate gyrus Neuroscience 2007 148 593 598 10.1016/j.neuroscience.2007.06.040 17706367

[B31] Forger NG Cell death and sexual differentiation of the nervous system Neuroscience 2006 138 929 938 10.1016/j.neuroscience.2005.07.006 16310316

[B32] Mong JA Glaser E McCarthy MM Gonadal steroids promote glial differentiation and alter neuronal morphology in the developing hypothalamus in a regionally specific manner J Neurosci 1999 19 1464 1472 9952422 10.1523/JNEUROSCI.19-04-01464.1999 PMC6786024

[B33] Poulet FM Roessler ML Vancutsem PM Initial uterine alterations caused by developmental exposure to tamoxifen Reprod Toxicol 1997 11 815 822 10.1016/S0890-6238(97)00065-8 9407592

[B34] Yuan H Bowlby DA Brown TJ Hochberg RB MacLusky NJ Distribution of occupied and unoccupied estrogen receptors in the rat brain: effects of physiological gonadal steroid exposure Endocrinology 1995 136 96 105 10.1210/en.136.1.96 7828562

[B35] Shughrue PJ Lane MV Merchenthaler I Regulation of progesterone receptor messenger ribonucleic acid in the rat medial preoptic nucleus by estrogenic and antiestrogenic compounds: an in situ hybridization study Endocrinology 1997 138 5476 5484 10.1210/en.138.12.5476 9389534

[B36] Galea LA Gonadal hormone modulation of neurogenesis in the dentate gyrus of adult male and female rodents Brain Res Rev 2008 57 332 341 10.1016/j.brainresrev.2007.05.008 17669502

[B37] Taupin P BrdU immunohistochemistry for studying adult neurogenesis: paradigms, pitfalls, limitations, and validation Brain Res Rev 2007 53 198 214 10.1016/j.brainresrev.2006.08.002 17020783

[B38] Norbury C Nurse P Animal cell cycles and their control Annu Rev Biochem 1992 61 441 470 10.1146/annurev.bi.61.070192.002301 1497317

[B39] Lopez F Belloc F Lacombe F Dumain P Reiffers J Bernard P Boisseau MR Modalities of synthesis of Ki67 antigen during the stimulation of lymphocytes Cytometry 1991 12 42 49 10.1002/cyto.990120107 1999122

[B40] Endl E Gerdes J The Ki-67 protein: fascinating forms and an unknown function Exp Cell Res 2000 257 231 237 10.1006/excr.2000.4888 10837136

[B41] Zacchetti A van Garderen E Teske E Nederbragt H Dierendonck JH Rutteman GR Validation of the use of proliferation markers in canine neoplastic and non-neoplastic tissues: comparison of KI-67 and proliferating cell nuclear antigen (PCNA) expression versus in vivo bromodeoxyuridine labelling by immunohistochemistry APMIS 2003 111 430 438 10.1034/j.1600-0463.2003.t01-1-1110208.x 12752223

[B42] Scholzen T Gerdes J The Ki-67 protein: from the known and the unknown J Cell Physiol 2000 182 311 322 10.1002/(SICI)1097-4652(200003)182:3<311::AID-JCP1>3.0.CO;2-9 10653597

[B43] Barha CK Lieblich SE Galea LA Different forms of oestrogen rapidly upregulate cell proliferation in the dentate gyrus of adult female rats J Neuroendocrinol 2009 21 155 166 10.1111/j.1365-2826.2008.01809.x 19076272

[B44] Galea LA Spritzer MD Barker JM Pawluski JL Gonadal hormone modulation of hippocampal neurogenesis in the adult Hippocampus 2006 16 225 232 10.1002/hipo.20154 16411182

[B45] Woolley CS Estrogen-mediated structural and functional synaptic plasticity in the female rat hippocampus Horm Behav 1998 34 140 148 10.1006/hbeh.1998.1466 9799624

[B46] Lee SJ Romeo RD Svenningsson P Campomanes CR Allen PB Greengard P McEwen BS Estradiol affects spinophilin protein differently in gonadectomized males and females Neuroscience 2004 127 983 988 10.1016/j.neuroscience.2004.05.049 15312910

[B47] Woolley CS Weiland NG McEwen BS Schwartzkroin PA Estradiol increases the sensitivity of hippocampal CA1 pyramidal cells to NMDA receptor-mediated synaptic input: correlation with dendritic spine density J Neurosci 1997 17 1848 1859 9030643 10.1523/JNEUROSCI.17-05-01848.1997 PMC6573364

[B48] Woolley CS McEwen BS Estradiol regulates hippocampal dendritic spine density via an N-methyl-D-aspartate receptor-dependent mechanism J Neurosci 1994 14 7680 7687 7996203 10.1523/JNEUROSCI.14-12-07680.1994 PMC6576901

[B49] Leranth C Petnehazy O MacLusky NJ Gonadal hormones affect spine synaptic density in the CA1 hippocampal subfield of male rats J Neurosci 2003 23 1588 1592 12629162 10.1523/JNEUROSCI.23-05-01588.2003 PMC6741990

[B50] Barker JM Galea LA Repeated estradiol administration alters different aspects of neurogenesis and cell death in the hippocampus of female, but not male, rats Neuroscience 2008 152 888 902 10.1016/j.neuroscience.2007.10.071 18353559

[B51] Levin ER Cell localization, physiology, and nongenomic actions of estrogen receptors J Appl Physiol 2001 91 1860 1867 11568173 10.1152/jappl.2001.91.4.1860

[B52] Dahlman-Wright K Cavailles V Fuqua SA Jordan VC Katzenellenbogen JA Korach KS Maggi A Muramatsu M Parker MG Gustafsson JA International Union of Pharmacology. LXIV. Estrogen receptors Pharmacol Rev 2006 58 773 781 10.1124/pr.58.4.8 17132854

[B53] Isgor C Watson SJ Estrogen receptor alpha and beta mRNA expressions by proliferating and differentiating cells in the adult rat dentate gyrus and subventricular zone Neuroscience 2005 134 847 856 10.1016/j.neuroscience.2005.05.008 15994024

[B54] Mazzucco CA Lieblich SE Bingham BI Williamson MA Viau V Galea LA Both estrogen receptor alpha and estrogen receptor beta agonists enhance cell proliferation in the dentate gyrus of adult female rats Neuroscience 2006 141 1793 1800 10.1016/j.neuroscience.2006.05.032 16797852

[B55] Mitra SW Hoskin E Yudkovitz J Pear L Wilkinson HA Hayashi S Pfaff DW Ogawa S Rohrer SP Schaeffer JM McEwen BS Alves SE Immunolocalization of estrogen receptor beta in the mouse brain: comparison with estrogen receptor alpha Endocrinology 2003 144 2055 2067 10.1210/en.2002-221069 12697714

[B56] Ivanova T Beyer C Ontogenetic expression and sex differences of aromatase and estrogen receptor-alpha/beta mRNA in the mouse hippocampus Cell Tissue Res 2000 300 231 237 10.1007/s004410000199 10867819

[B57] Shughrue PJ Lane MV Merchenthaler I Comparative distribution of estrogen receptor-alpha and -beta mRNA in the rat central nervous system J Comp Neurol 1997 388 507 525 10.1002/(SICI)1096-9861(19971201)388:4<507::AID-CNE1>3.0.CO;2-6 9388012

[B58] Prewitt AK Wilson ME Changes in estrogen receptor-alpha mRNA in the mouse cortex during development Brain Res 2007 1134 62 69 10.1016/j.brainres.2006.11.069 17207781 PMC3443600

[B59] Miranda RC Toran-Allerand CD Developmental expression of estrogen receptor mRNA in the rat cerebral cortex: a nonisotopic in situ hybridization histochemistry study Cereb Cortex 1992 2 1 15 10.1093/cercor/2.1.1 1633405

[B60] Solum DT Handa RJ Localization of estrogen receptor alpha (ER alpha) in pyramidal neurons of the developing rat hippocampus Brain Res Dev Brain Res 2001 128 165 175 10.1016/S0165-3806(01)00171-7 11412902

[B61] Martinez-Cerdeno V Noctor SC Kriegstein AR Estradiol stimulates progenitor cell division in the ventricular and subventricular zones of the embryonic neocortex Eur J Neurosci 2006 24 3475 3488 10.1111/j.1460-9568.2006.05239.x 17229096

[B62] Konkle AT McCarthy MM Developmental Time Course of Estradiol, Testosterone, and Dihydrotestosterone Levels in Discrete Regions of Male and Female Rat Brain Endocrinology 2010 21068160 10.1210/en.2010-0607 PMC3033055

[B63] Lam TT Leranth C Role of the medial septum diagonal band of Broca cholinergic neurons in oestrogen-induced spine synapse formation on hippocampal CA1 pyramidal cells of female rats Eur J Neurosci 2003 17 1997 2005 10.1046/j.1460-9568.2003.02637.x 12786965

[B64] Campbell NR Fernandes CC Halff AW Berg DK Endogenous signaling through alpha7-containing nicotinic receptors promotes maturation and integration of adult-born neurons in the hippocampus J Neurosci 2010 30 8734 8744 10.1523/JNEUROSCI.0931-10.2010 20592195 PMC2905643

[B65] Mitsushima D Takase K Takahashi T Kimura F Activational and organisational effects of gonadal steroids on sex-specific acetylcholine release in the dorsal hippocampus J Neuroendocrinol 2009 21 400 405 10.1111/j.1365-2826.2009.01848.x 19356199

[B66] Mitsushima D Takase K Funabashi T Kimura F Gonadal steroids maintain 24 h acetylcholine release in the hippocampus: organizational and activational effects in behaving rats J Neurosci 2009 29 3808 3815 10.1523/JNEUROSCI.5301-08.2009 19321777 PMC6665029

[B67] Schaevitz LR Berger-Sweeney J Neurogenesis of the cholinergic medial septum in female and male C57BL/6J mice J Neurobiol 2005 65 294 303 10.1002/neu.20188 16187329

[B68] Crews JK Murphy JG Khalil RA Gender differences in Ca(2+) entry mechanisms of vasoconstriction in Wistar-Kyoto and spontaneously hypertensive rats Hypertension 1999 34 931 936 10523387 10.1161/01.hyp.34.4.931

[B69] Murphy JG Khalil RA Gender-specific reduction in contractility and [Ca(2+)](i) in vascular smooth muscle cells of female rat Am J Physiol Cell Physiol 2000 278 C834 844 10751331 10.1152/ajpcell.2000.278.4.C834

[B70] Li Z Krause DN Doolen S Duckles SP Ovariectomy eliminates sex differences in rat tail artery response to adrenergic nerve stimulation Am J Physiol 1997 272 H1819 1825 9139968 10.1152/ajpheart.1997.272.4.H1819

[B71] Geary GG Krause DN Duckles SP Estrogen reduces mouse cerebral artery tone through endothelial NOS- and cyclooxygenase-dependent mechanisms Am J Physiol Heart Circ Physiol 2000 279 H511 519 10924048 10.1152/ajpheart.2000.279.2.H511

[B72] Haynes MP Sinha D Russell KS Collinge M Fulton D Morales-Ruiz M Sessa WC Bender JR Membrane estrogen receptor engagement activates endothelial nitric oxide synthase via the PI3-kinase-Akt pathway in human endothelial cells Circ Res 2000 87 677 682 11029403 10.1161/01.res.87.8.677

[B73] Stirone C Boroujerdi A Duckles SP Krause DN Estrogen receptor activation of phosphoinositide-3 kinase, akt, and nitric oxide signaling in cerebral blood vessels: rapid and long-term effects Mol Pharmacol 2005 67 105 113 10.1124/mol.104.004465 15496504

[B74] Chambliss KL Yuhanna IS Mineo C Liu P German Z Sherman TS Mendelsohn ME Anderson RG Shaul PW Estrogen receptor alpha and endothelial nitric oxide synthase are organized into a functional signaling module in caveolae Circ Res 2000 87 E44 52 11090554 10.1161/01.res.87.11.e44

[B75] Pare G Krust A Karas RH Dupont S Aronovitz M Chambon P Mendelsohn ME Estrogen receptor-alpha mediates the protective effects of estrogen against vascular injury Circ Res 2002 90 1087 1092 10.1161/01.RES.0000021114.92282.FA 12039798

[B76] Mendelsohn ME Genomic and nongenomic effects of estrogen in the vasculature Am J Cardiol 2002 90 3F 6F 10.1016/S0002-9149(02)02418-9 12106632

[B77] Trejo JL Carro E Torres-Aleman I Circulating insulin-like growth factor I mediates exercise-induced increases in the number of new neurons in the adult hippocampus J Neurosci 2001 21 1628 1634 11222653 10.1523/JNEUROSCI.21-05-01628.2001 PMC6762955

[B78] van Praag H Kempermann G Gage FH Running increases cell proliferation and neurogenesis in the adult mouse dentate gyrus Nat Neurosci 1999 2 266 270 10.1038/6368 10195220

[B79] Altman J Das GD Postnatal neurogenesis in the guinea-pig Nature 1967 214 1098 1101 10.1038/2141098a0 6053066

[B80] Gould E Tanapat P McEwen BS Flugge G Fuchs E Proliferation of granule cell precursors in the dentate gyrus of adult monkeys is diminished by stress Proc Natl Acad Sci USA 1998 95 3168 3171 10.1073/pnas.95.6.3168 9501234 PMC19713

[B81] Moore CL The role of maternal stimulation in the development of sexual behavior and its neural basis Ann N Y Acad Sci 1992 662 160 177 10.1111/j.1749-6632.1992.tb22859.x 1456637

[B82] Knable MB Barci BM Webster MJ Meador-Woodruff J Torrey EF Molecular abnormalities of the hippocampus in severe psychiatric illness: postmortem findings from the Stanley Neuropathology Consortium Mol Psychiatry 2004 9 609 620 544 10.1038/sj.mp.4001471 14708030

[B83] McEwen BS Possible mechanisms for atrophy of the human hippocampus Mol Psychiatry 1997 2 255 262 10.1038/sj.mp.4000254 9152991

[B84] Nestor PG Kubicki M Kuroki N Gurrera RJ Niznikiewicz M Shenton ME McCarley RW Episodic memory and neuroimaging of hippocampus and fornix in chronic schizophrenia Psychiatry Res 2007 155 21 28 10.1016/j.pscychresns.2006.12.020 17395435

[B85] Tanskanen P Veijola JM Piippo UK Haapea M Miettunen JA Pyhtinen J Bullmore ET Jones PB Isohanni MK Hippocampus and amygdala volumes in schizophrenia and other psychoses in the Northern Finland 1966 birth cohort Schizophr Res 2005 75 283 294 10.1016/j.schres.2004.09.022 15885519

[B86] Schmidt-Kastner R Freund TF Selective vulnerability of the hippocampus in brain ischemia Neuroscience 1991 40 599 636 10.1016/0306-4522(91)90001-5 1676492

